# The Frequency and Importance of Common α-globin Gene Deletions Among β-Thalassemia Carriers in an Iranian Population

**Published:** 2018

**Authors:** Azam Moosavi, Ali M. Ardekani

***Avicenna J Med Biotech 2017; 9(4): 196–200***

The Editorial Board of *Avicenna Journal of Medical Biotechnology (AJMB)* has decided to retract the original article entitled “The Frequency and Importance of Common α-globin Gene Deletions Among β-Thalassemia Carriers in an Iranian Population” published in the October–December 2017 issue due to a fact which is contrary to the scientific rules and regulation of AJMB.

